# Diagnosis of ABCB11 gene mutations in children with intrahepatic cholestasis using high resolution melting analysis and direct sequencing

**DOI:** 10.3892/mmr.2014.2349

**Published:** 2014-06-20

**Authors:** GUORUI HU, PING HE, ZHIFENG LIU, QIAN CHEN, BIXIA ZHENG, QIHUA ZHANG

**Affiliations:** 1 Medical College of Nanjing University, Nanjing, Jiangsu 210093, P.R. China; 2 Department of Digestive Disease, Nanjing Children’s Hospital, Nanjing Medical University, Nanjing, Jiangsu 210008, P.R. China

**Keywords:** ABCB11, intrahepatic cholestasis, high resolution melting analysis, single nucleotide polymorphisms

## Abstract

Intrahepatic cholestasis represents a heterogeneous group of disorders that begin during childhood, most commonly manifesting as neonatal cholestasis, and lead to ongoing liver dysfunction in children and adults. For children, inherited pathogenic factors of cholestasis have gained increasing attention owing to the rapid development of molecular biology technology. However, these methods have their advantages and disadvantages in terms of simplicity, sensitivity, specificity, time required and expense. In the present study, an effective, sensitive and economical method is recommended, termed high-resolution melting (HRM) analysis and direct sequencing, based on general polymerase chain reaction, to detect mutations in disease-causing genes. As one type of inherited intrahepatic cholestasis, progressive familial intrahepatic cholestasis type 2 (PFIC2) is caused by pathogenic mutations in the ABCB11 gene, HRM was used to detect mutations in the ABCB11 gene in the present study, and the diagnosis for PFIC2 was made by comprehensive analysis of genetic findings and clinical features. Furthermore, the characteristics of mutations and single nucleotide polymorphisms (SNPs) in the ABCB11 gene were elucidated. A total of 14 types of mutations/polymorphisms were identified in 20 patients from mainland China, including six missense mutations (p.Y337H, p.Y472C, p.R696W, p.Q931P, p.D1131V and p.H1198R), one nonsense mutation (p.R928X) and seven SNPs (p.D36D/rs3815675, p.F90F/rs4148777, p.Y269Y/rs2287616, p.I416I/rs183390670, p.V444A/rs2287622, p.A865V/rs118109635 and p.A1028A/rs497692). Five mutations were novel. The majority of the mutations were different from those detected in other population groups. A total of 4/20 patients (1/5) were diagnosed to be PFIC2 by combining genetic findings with the clinical features. Polymorphisms V444A and A1028A, with an allele frequency of 74.5 and 67.2%, respectively, were highly prevalent in the mainland Chinese subjects. No differences were found between the patients with cholestasis and the control subjects. Efficient genetic screening facilitates the clinical diagnosis of genetic disorders. The present study demonstrated that HRM analysis was efficient and effective in detecting mutations and expanded the known spectrum of ABCB11 gene mutations.

## Introduction

Cholestasis, one of the most common presentations of liver disease, is the major cause of severe liver dysfunction in childhood, which eventually necessitates liver transplantation (1). Despite extensive investigation, the etiology and pathogenesis of cholestasis in a significant proportion of children remains unknown. Recent advances in molecular biology have elucidated the genetic basis for a subgroup of disorders, including progressive familial intrahepatic cholestasis (PFIC). PFIC is a heterogeneous group of autosomal recessive disorders characterized by early onset cholestasis, progressive liver cirrhosis and hepatic failure in early childhood (2). According to the genetic defects, three types of PFIC have been identified. PFIC1, PFIC2 and PFIC3 are caused by mutations in ATP8B1 (encoding FIC1), ABCB11 [encoding bile salt export pump, (BSEP)] and ABCB4 [encoding multidrug resistant protein 3, (MDR3)] genes, respectively (3). Although the actual prevalence of PFIC remains unknown, the estimated incidence is 1/50,000–100,000 births worldwide (4).

The ABCB11 gene located on chromosome 2q24 encodes an ATP-binding cassette (ABC) transporter, known as BSEP in human liver. BSEP is selectively expressed in the hepatocyte canalicular membrane and is the major exporter of bile salts against extreme concentration gradients, in an ATP-dependent manner (5). Mutations in this gene are responsible for decreased biliary bile salt secretion, which leads to decreased bile flow and the accumulation of bile salts inside the hepatocytes, thereby resulting in a spectrum of cholestatic disease (6). This ranges from the severe phenotype of PFIC2 (7) to milder, intermittent forms of cholestasis, including benign recurrent intrahepatic cholestasis type 2 (8), drug-induced cholestasis (DIC) (9), intrahepatic cholestasis pregnancy (ICP) (10) and contraceptive-induced cholestasis (11). Furthermore, two significant single nucleotide polymorphisms (SNPs) of ABCB11 have been associated with non-cholestatic and cholestatic liver disease. One is V444A (HGVS name: NM_003742.2:c.1331T>C; refSNP: rs2287622) in exon 13, which has been reported to be correlated with cholestasis (12) and chronic hepatititis C virus infection (13). Furthermore, the polymorphism was able to reduce the levels of mature BSEP (14). The other is A1028A (HGVS name: NM_003742.2:c.3084A>G; refSNP: rs497692) in exon 24. It has been described to cause altered splicing events of ABCB11 through severe exon skipping *in vitro* (14) and possibly associates with primary biliary cirrhosis (PBC) (15). Whether they are associated with cholestatic liver disease; however, remains controversial (11,16).

Clinical and biochemical characteristics of patients with ABCB11 mutations in early infancy, include jaundice, pruritus, growth failure, hepatomegaly, splenomegaly, complications due to fat-soluble vitamin deficiency, increased serum bilirubin concentrations particularly direct bilirubin, normal or low serum γ-glutamyltransferase (GGT) and high serum bile acid concentrations (6). These symptoms are not specific and often overlap with those of other cholestatic liver diseases, such as biliary atresia, inherited cholestatic disease, neonatal hepatitis and metabolic diseases, rendering prompt and accurate clinical diagnosis particularly difficult. Previously, mutations in ABCB11 have been detected predominantly in European and American patients. Studies involving Asian patients, particularly Chinese subjects, are notably lacking (16,17). Current diagnostic methods used for inherited intrahepatic cholestasis include clinical features, biochemical parameters, liver histology and genetic analysis. These lack proficient accuracy, are time consuming and expensive or are not widely available. High-resolution melting (HRM) analysis prior to sequencing has been described as an effective, sensitive and economical method for detecting genetic variations (18).

The present study aimed to apply HRM analysis prior to sequencing to identify genetic variations more efficiently in the ABCB11 gene in Chinese patients. This screening approach allows a faster and more economical diagnosis to be made in patients suspected of carrying ABCB11 mutations.

## Materials and methods

### Patients and DNA samples

All of the subjects were recruited prospectively at the Nanjing Children’s Hospital Affiliated to Nanjing Medical University (Nanjing, China) between July 2010 and June 2013. All patients were <1 year old and suffered from cholestasis, presenting with elevated conjugated hyperbilirubinaemia or pruritus. Basic demographic data of the patients and blood biochemistry examinations were reviewed from the medical records. A total of 20 patients (numbered P1–P20) were included for detection of ABCB11 mutations. The inclusion criteria were as follows: (i) Onset of conjugated hyperbilirubinaemia or pruritus prior to 12 months of age. Conjugated hyperbilirubinaemia was defined as a direct bilirubin level of 20% of the total bilirubin or >17.1 μmol/l if the total serum bilirubin was <85.5 μmol/l (17); (ii) the serum GGT levels were consistently below the normal value for infancy (<94 u/l) (19); (iii) clinical manifestations, including jaundice, pruritus, growth failure, hepatomegaly or splenomegaly were observed and (iv) a thorough search for the known causes of cholestasis in infancy had been negative, including viral or bacterial infection and inborn errors of bile acid synthesis. Patients with biliary tree anomalies were excluded.

All of the DNA samples were extracted from peripheral EDTA-anticoagulated whole blood of all the patients and some parents using a TIANamp Blood DNA kit (Tiangen Biotech (Beijing) Co., Ltd, Beijing, China), according to the manufacturer’s instructions. Informed consent was obtained from all of the parents of the infants involved in the study. The study was approved by the Institutional Review Board of Nanjing Children’s Hospital Affiliated to Nanjing Medical University (Nanjing, China).

### Polymerase chain reaction (PCR) amplification of exons of ABCB11

A total of 27 sets of primers were designed to obtain amplicons with a size ≤400 bp (range, 113–321) for the coding sequences and splice sites (ten nucleotides away from the exon) of exon 2–28 of the ABCB11 gene (Table I). The concentrations of DNA samples were measured using the NanoDrop 2000 UV-Vis Spectrophotometer (Thermo Scientific, Wilmington, DE, USA), then the DNA samples were diluted to 10 ng/μl. PCR was performed in a total volume of 10 μl containing 30 ng genomic DNA, 3 pmol of each primer, 1 μl of 10X *Taq* Buffer with (NH_4_)_2_SO_4_, 1 μl of dNTP Mixture (2.5 mM each), 1 μl of 25 mM MgCl_2_, 0.4 units *Taq* DNA Polymerase (recombinant; Thermo Scientific), 1 μl of 1X LCGreen^®^ PLUS (Idaho Technology, Salt Lake City, UT, USA) and water (molecular grade) added up to 10 μl. A PTC-200 DNA Engine^®^ Peltier Thermo Cycler (Bio-Rad, Hercules, CA, USA) was used for PCR with the following cycling conditions: An initial denaturation step at 95°C for 5 min, followed by 40 cycles consisting of denaturation at 95°C for 30 sec, annealing at 61°C for 30 sec, extension at 72°C for 20 sec and a final extension step at 72°C for 10 min followed by cooling to 25°C for 5 min.

### HRM analysis

Following completion of PCR, 10 μl of the PCR products were added into a 96-well plate (Bio-Rad). The mixtures were overlaid with 20 μl of mineral oil (Sigma-Aldrich, St. Louis, MO, USA) and the plate was centrifuged at 2,000 × g for 1 min. The plate was transferred to the LightScanner^®^ (Idaho Technology), with fluorescence data collection over the temperature range 70–97°C, as samples were melted.

In all instances, HRM directly discriminates the heterozygotes and homozygotes, but not the major and minor homozygotes of a polymorphism. Next, genotyping using spike-in control DNA was performed to allow distinction of minor homozygotes from major homozygotes. Briefly, the PCR products that were demonstrated to be homozygous were mixed with an equal quantity of DNA products from a known major allele homozygous subject to apply to the LightScanner. To promote heteroduplex formation, the mixtures were firstly centrifuged at 2,000 × g for 1 min, then denatured in a PTC-100^®^ Peltier Thermo Cycler (Bio-Rad) under the following conditions: 95°C for 2 min, 25°C for 1 min, 95°C for 2 min, 25°C for 2 min. This strategy converted the minor allele homozygous form into the heterozygous form, but did not change the major allele homozygous samples, providing the distinction between the major allele homozygous samples and the minor ones. Each sample was analyzed at least three times and all of the samples demonstrated reproducible results.

### Direct sequencing

The samples with shifted melting curves, as identified by HRM, were amplified through another PCR reaction with different primer pairs to achieve ~500-bp PCR products for direct sequencing (Table II). The reaction mixture in a total of 50 μl contained 50 ng of genomic DNA, 20 pmol of each primer, 5 μl of 10X Ex *Taq* Buffer (Mg^2+^), 4 μl dNTP mixture (2.5 mM each), 1 unit of Ex *Taq* DNA Polymerase (Hot Start; Takara Bio Inc., Dalian, China) and water (molecular grade) added up to 50 μl. The PCR reaction was performed under the following conditions: Initial denaturation at 95°C for 5 min; 40 cycles of 94°C for 30 sec, 59°C for 30 sec, 72°C for 40 sec and one cycle of 72°C for 10 min. The PCR products were separated on 2% agarose gels, purified with a gel extraction kit (Tiangen Biotech (Beijing) Co., Ltd). Direct sequencing of the purified products was performed in a 3130 Genetic analyzer (Applied Biosystems, Foster City, CA, USA) using an ABI BigDye^®^ Terminator v3.1 Cycle Sequencing kit (Applied Biosystems). All of the tests were performed in duplicate. The sequences obtained were compared with the ABCB11 reference sequence (NM_003742.2) derived from publicly available databases provided by NCBI (http://www.ncbi.nlm.nih.gov/).

To avoid false-negative results from HRM screening, confirmative direct sequencing was performed for exons 13 and 24 in which SNPs V444A (in exon 13) and A1028A (in exon 24) were common and rendered the interpretation of melting patterns difficult. For the remaining 25 exons, 250 amplicons (50%) without melting curve shifting were randomly selected for direct sequencing. In total, the results of 290/540 amplicons from HRM analysis were confirmed by direct sequencing.

### Prediction of functional consequences

The variants confirmed by direct sequencing were firstly submitted to the MutationTaster program to evaluate the disease-causing potential of sequence alterations (http://www.mutationtaster.org/) (20). To predict the functional consequences of the missense mutations and polymorphisms identified, three bioinformatics tools based on different computational methods were applied. Sorting Intolerant From Tolerant (SIFT) is a program that predicts whether an amino acid substitution affects protein function, through analysis of an alignment of orthologous sequences (http://sift.jcvi.org/) (21). A SIFT score of <0.05 indicates the presence of evolutionarily conserved amino acids, and mutations in these residues are predicted to be deleterious (22). Polymorphism Phenotyping version 2 (PolyPhen-2) uses empirically derived rules to predict the possible impact of an amino acid substitution on the structure and function of a human protein (http://genetics.bwh.harvard.edu/pph2/) (23). It calculates a position-specific independent count (PSIC) profile for each candidate mutation and qualitatively predicts whether it is benign, possibly damaging or highly likely damaging, according to the posterior probability intervals (0, 0.2), (0.2, 0.85) and (0.85, 1), respectively (22). SNPs&GO characterized by statistical accuracy is a web server for the prediction of human disease-related single point protein mutations, by collecting a unique framework of information derived from protein sequences, protein sequence profiles and protein function (http://snps-and-go.biocomp.unibo.it/snps-and-go/) (24). The mutations effecting amino acid sequence were estimated to be neutral or disease-related. The amino acid changes were also classified as evolutionarily conserved (EC) or non-conserved (EN), based on sequence alignments with six mammalian orthologs (bonobo, mouse, rat, rabbit, dog, cattle). It has been demonstrated that substitutions at evolutionarily conserved positions are more deleterious than those at evolutionarily non-conserved positions (25). In order to understand the prediction of functional consequences, the schematic view of BESP with the location of the variants identified in the present study was produced using the TOPO program (http://www.sacs.ucsf.edu/TOPO2/).

### Analysis of mutation carrier rate in control subjects

To determine the carrier rate of mutations identified, the exons in which mutations existed were screened using HRM analysis of 200 control subjects from the visitors to the Nanjing Children’s Hospital Affiliated to Nanjing Medical University (Nanjing, China). All of the controls were ≤1 year old and had normal liver and biliary function. All of the exons with abnormal melting patterns and 50% of the exons with normal melting patterns were confirmed by direct sequencing.

### Analysis of V444A and A1028A in patients and controls

To investigate whether the two common polymorphisms found in the study population have functional consequences and affect disease presentation, the allele frequencies of polymorphisms were analyzed in 20 patients and in the 200 control subjects mentioned above. These samples were tested using HRM analysis of exons 13 and 24, followed by direct sequencing to identify the V444A and A1028A polymorphisms. Statistical analyses were performed using Stata 10 (Stata Corporation, College Station, TX, USA). Associations of categorical variables were tested by Pearson’s χ^2^ test. Allele frequencies were tested to determine whether they were in Hardy-Weinberg equilibrium. All tests were two-sided and P<0.017 was considered to indicate a statistically significant difference according to the Bonferroni correction for multiple testing.

## Results

### Mutations and SNPs detected in patients

**Figure 1:**
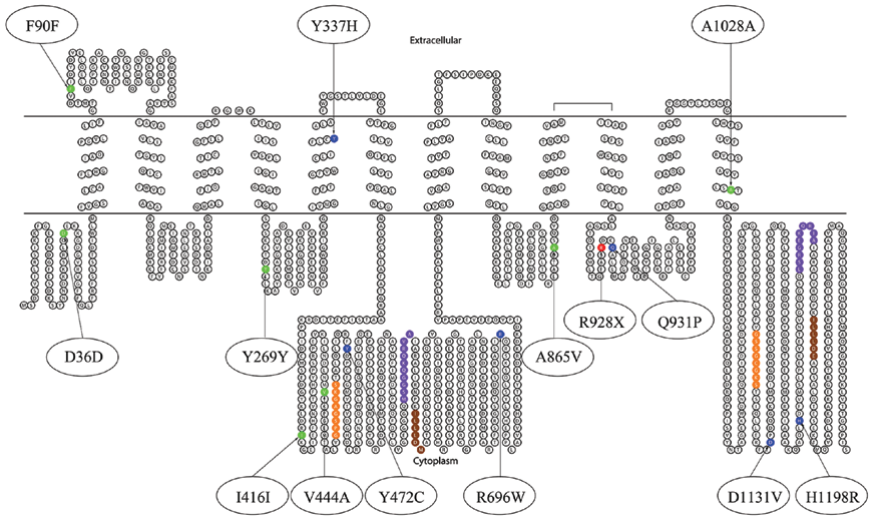
Schematic view of BSEP (NCBI reference sequence, NP_003733.2) with the location of the variants identified in the study using the TOPO2 program (http://www.sacs.ucsf.edu/TOPO2/). Walker A motif, ABC transporter signature motif and Walker B motif (33) are illustrated in orange, purple and brown, respectively. The genetic variants are represented as green for single nucleotide polymorphism, blue for missense mutation and red for nonsense mutation.

**Figure 2:**
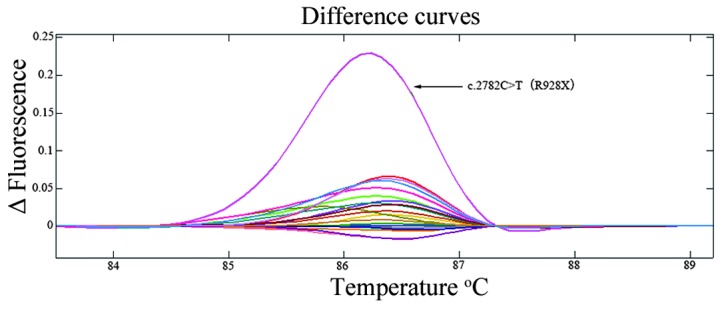
Examples of high-resolution melting analysis for screening mutations/single nucleotide polymorphisms of the ABCB11 gene. In difference plots, the melting profile of a wild-type control was selected as a horizontal base line and the relative differences in the melting of all the other samples were plotted relative to this baseline. In exon 22, the melting curve of the patient with heterozygous p.R928X (c.2782C>T) shifted away from those without mutations or with homozygous mutations. The strategy of spike-in control DNA was used and the pattern of melting curves did not change. Following direct sequencing, p.R928X was identified.

Among the 20 patients with cholestasis, 14 types of variants were detected, including seven mutations in the coding region (p.Y337H, p.Y472C, p.R696W, p.R928X, p.Q931P, p.D1131V and p.H1198R) and seven SNPs (p.D36D/rs3815675, p.F90F/rs4148777, p.Y269Y/rs2287616, p.I416I/rs183390670, p.V444A/rs2287622, p.A865V/rs118109635 and p.A1028A/rs497692). Missense mutations p.Y337H, p.R696W, p.Q931P, p.D1131V and p.H1198R were novel mutations identified in the study. All of the mutations and SNPs identified in the study are summarized in Table III and Fig. 1. The shifted melting curves of mutated samples are presented in Fig. 2.

The results based on comprehensive evaluation of SIFT, PolyPhen-2, SNPs&GO and evolution conservation indicated that p.Y337H, p.Y472C, p.R696W, p.D1131V and p.H1198R were likely damaging, p.Q931P and p.A865V were possibly damaging and p.V444A was predicted to be benign. With the nonsense mutation, p.R928X is able to introduce a premature stop codon that results in premature protein truncation or failure of protein production, which had been reported previously in PFIC2 patients (26). Four patients (P1, P3, P5 and P11) were identified to have PFIC2 based on the comprehensive consideration of clinical features and genetic analysis. The consequences of function prediction of missense mutations and polymorphisms for BSEP are demonstrated in Table IV.

Among the four patients with disease-causing mutations (P1, P3, P5 and P11), two were heterozygous and two exhibited compound heterozygous mutations (Table V). Except for P5 and P11 whose parents’ samples were not available, the parents of P1 and P3 carried one allele of the respective mutation each. The two SNPs of p.V444A and p.A1028A were also detected in the four patients, except that P11 only had p.V444A. The detection rate of disease-causing mutations in ABCB11 was 4/20 (20%) patients from different families.

### Rates of false-positive calls and false-negative calls by HRM analysis

Among the 540 amplicons from the 20 patients, 58 amplicons demonstrated melting curve shifting and 56 (96.6%) of these samples were found to have mutations by direct sequencing. The false-positive call rate was 0.37% (2/540) by HRM analysis. As for the rates of false-negative calls, no mutations or polymorphisms detected from direct sequencing were missed by the HRM analysis of the 290 amplicons consisting of the two exons with common SNPs in the Chinese population (exons 13 and 24) and the randomly selected 250 amplicons from the remaining 25 exons that did not demonstrate melting curve shifting. The false-negative rate was zero.

### Mutation carrier rate in control subjects

All the disease-related mutations detected above were tested in 200 control subjects using HRM analysis to screen exon 10 (p.Y337H), exon 13 (p.Y472C), exon 18 (p.R696W), exon 22 (p.R928X), exon 22 (p.Q931P), exon 25 (p.D1131V) and exon 26 (p.H1198R). No distinct mutation patterns were noted in all of the 200 control subjects.

### Polymorphism analysis

Two previously reported SNPs, p.V444A and p.A1028A, were identified in a number of the patients and control subjects. These two polymorphic sites were examined with HRM analysis followed by direct sequencing in four patients with PFIC2, 16 patients with cholestasis of undefined etiology from the 20 patients who were not PFIC2, and 200 control patients. The distribution of p.V444A and p.A1028A polymorphisms, as well as allele frequencies, are revealed in Table VI. It was identified that V444A and A1028A were more prevalent polymorphisms in the control subjects, and had an allele frequency of 74.5 and 67.2%, respectively. This was consistent with previously reported data, which identified an allele frequency of 75.6% for V444A (19). However, neither V444A nor A1028A were associated with PFIC2 or cholestasis of undefined etiology in the patients. The distribution of alleles at the two SNPs in control group was in Hardy-Weinberg equilibrium (V444A, P=0.46; A1028A, P=0.43).

## Discussion

Numerous PCR-based mutation detection methods have been used to screen ABCB11 mutations. Methodologies described in the literature include the use of single-strand conformation polymorphisms (27), denaturing high performance liquid chromatography (19), gene chips (28), TaqMan probes (29), microsatellite markers (27), restriction fragment length polymorphisms (27) and DNA sequencing (17). All of these methods have their advantages and disadvantages in terms of simplicity, sensitivity, specificity, time required and expense.

Direct sequencing is considered to be the standard method in nucleic acids studies, as it is able to directly identify the specific mutations that may be present. However, it has the disadvantages of high cost, long turnaround time and weak sensitivity, limiting its practicality in diagnostic settings (30). Therefore, the present study attempted to perform HRM analysis, prior to sequencing, for screening ABCB11 mutations in order to achieve high affectivity and efficiency, as well as low cost.

HRM analysis is a powerful technique for the rapid detection of mutations in double-stranded DNA samples that has the potential to meet clinical demand (31). HRM involves precise monitoring of the change in fluorescence caused by the release of a saturating double-stranded DNA-binding fluorescent dye from a DNA duplex, which is denatured by increasing temperature. Sequence variants, including mutations and SNPs, are detected from the differences in the melting profiles between the test and reference DNA (32).

In the present study, HRM analysis exhibited marked affectivity and efficiency for detecting variants of the ABCB11 gene. It exhibited a high sensitivity (estimated 100% in this study), high speed (30 min for processing 96 samples concurrently) and low cost (the reagents used in the present study cost an estimated US $0.5/sample/amplicon). Furthermore, the false-negative rate was zero and the rate of false-positive calls, estimated to be 0.37%, was allowable as the mutations were further confirmed by direct sequencing.

Mutation detection in the ABCB11 gene is important in confirming the diagnosis of PFIC2. Among the seven mutations identified in the study, p.Y337H, p.R696W, p.Q931P, p.D1131V and p.H1198R are novel mutations according to data from The Human Gene Mutation Database (http://www.hgmd.cf.ac.uk/ac/index.php). Also, none of the mutations were detected in the control subjects. The missense mutation p.Y337H (c.1009T>C) occurred in the transmembrane domain (TMD) that led to an amino acid substitution from tyrosine to histidine at position 337 in BSEP. By comparing amino acids at this locus between species, tyrosine was evolutionarily conserved. While the TMDs form the pore and define substrate specificity (33), changes in TMDs impairs the binding of substrate. Therefore, the change may affect the protein features. Furthermore, the nucleotide change in ABCB11 (c.1009T>C) introduced a serine/arginine-rich splicing factor 6 (SRSF6) binding site, that has a role in constitutive splicing and modulates the selection of alternative splice sites according to the results of the ESEfinder3.0 software (http://rulai.cshl.edu/cgi-bin/tools/ESE3/esefinder.cgi). In addition, precursor mRNA (pre-mRNA) splicing may be disturbed (14). All of the other four mutations occurred in intracellular loops. These mutations were evolutionarily conserved and an comprehensive analysis of the results of SIFT, PolyPhen-2, SNPs&GO revealed that they may be deleterious with the exception of p.Q931P. Correlation with clinical and genetic findings indicated that these mutations may be functionally relevant. The missense mutation p.Y472C and nonsense mutation p.R928X, have been reported in PFIC2 patients of European populations and immunohistochemical staining for BSEP was undetectable (26–28,34). The previous studies are consistent with the present study, which demonstrates that comprehensive analysis of functional prediction is essential and useful. Although studies have demonstrated that missense mutations in ABCB11 may impair BSEP processing and function, or disrupt pre-mRNA splicing *in vitro* (14), further studies are required to elucidate their definite roles.

ABCB11 gene mutations in children have been identified in Asian populations, including in Thailand (35), India (36), Japan (37–39), Taiwan (16,40,41) and mainland China (17,42), the majority of which are different from those reported in other populations. Common mutations that have been reported in European populations, such as E297G and D482G, were not detected in Chinese subjects. Therefore, mutations in ABCB11 may be ethnicity-specific as hypothesized by Ananthanarayanan and Li (43).

V444A and A1028A are two highly prevalent polymorphisms. The allele frequency of V444A and A1028A has been reported in Japanese and Caucasian populations (44). According to the present study, the allele frequencies of V444A and A1028A were 74.5% and 67.2%, respectively, in mainland Chinese populations. V444A has previously been implicated in ICP and DIC with a higher allele frequency than normal controls suggesting that this polymorphism may become disease relevant in certain conditions, such as pregnancy and the use of ethinylestradiol and levonorgestrel (11,45). A1028A occurs in the TMD and it has been demonstrated that it caused severe exon skipping (14). However, as the number of patients tested in the present study is small, it is not conclusive whether V444A or A1028A has a role in PFIC2 or cholestasis. Further larger scale studies focusing on specific populations are warranted to fully elucidate their roles. As the frequency of the two SNPs is so high, sequencing directly for exon 13 and exon 24 in case of omission is recommended.

In conclusion, the present study has established a rapid genetic test that screens mutations and SNPs across the entire ABCB11 coding regions. No common mutations in the ABCB11 gene were found in the Chinese population. V444A and A1028A were two highly prevalent SNPs found in ABCB11 exons in the study population; however, whether they are associated with pediatric cholestatic diseases remains unclear. Further studies investigating these mutations may enrich the knowledge of the ABCB11 gene and its product, BSEP, and may therefore be beneficial to the personalized management of individual patients in the future.

## Figures and Tables

**Table I:** Primers for PCR amplification of ABCB11 for HRM analysis.

Exon	Primer sequence	Amplicon length (bp)
2	F: 5′ CTTTCGTTTGGCTACTTTGATTA 3′ R: 5′ TGTACAAGATGCAGTGAGG 3′	213
3	F: 5′ CGTTGCATTTTGTCATTATTATTAACT 3′ R: 5′ AAAGATGCTGCATTGTTGAA 3′	113
4	F: 5′ ATTGTATTGGAAAGGGTGGTC 3′ R: 5′ TATAGCTGCACACCCACT 3′	144
5	F: 5′ AGATGATCTCTGAACCCTT 3′ R: 5′ TTGAATTACAATTTAAGATATGAGCAA 3′	329
6	F: 5′ CAAGTCTGAACATTCTTTTCCC 3′ R: 5′ CACATTGCATCTCATTGTAGTG 3′	191
7	F: 5′ TGCAATGCTAAACATTCCTT3′ R: 5′ AAGGGTTTTATTATCCAAAAATCAC3′	223
8	F: 5′ ACTGAGACTTTCAGCAAGATA 3′ R: 5′ CCCATGGAGAGATGCAA 3′	274
9	F: 5′ CTTACCTAATTTCTTGGACTTCACA 3′ R: 5′ ACTATGCTGATTGATGAAATTAAGGA 3′	228
10	F: 5′ TAACTTGAGCTGTTTCTGCC 3′ R: 5′ ATGTCTCGGTCAATAAGTCC 3′	252
11	F: 5′ CATGGAAGACCCAAATGATAGT 3′ R: 5′ CCCCACCTGTTAATGGC 3′	233
12	F: 5′ TAGTTTGAGTTTACACTGTGTCC 3′ R: 5′ ATCATCGAAGAAGAAAACATTTACTAT 3′	204
13	F: 5′ GTGACAATCTGAACTTTGCT 3′ R: 5′ TGATCAATATACTGCCATTTGC 3′	231
14	F: 5′ GTTGTGATGTTGTGCCC 3′ R: 5′ TTCCTTCTATGACCTCTTAGTTT 3′	282
15	F: 5′ CAGAAGCCATCAAATTCTTT 3′ R: 5′ GCATTTCCACATGGACC 3′	268
16	F: 5′ TTTGTTTAATGGTGCACTGT 3′ R: 5′ AAACCGTAAAGCACTATAGAC 3′	301
17	F: 5′ CTTGGATATGGTTCTGTTTATTGTA 3′ R: 5′ AAAGCTTGTAATCTGCCC 3′	157
18	F: 5′ GTCTACACTGTTTCATCTTTCTG 3′ R: 5′ CAGGAGAGACTTCTTCCATTC 3′	184
19	F: 5′ ATGTCTTGAGTACATTTAGATGAT 3′ R: 5′ TGAGAAGAAGAAAGCTAGTCCA 3′	249
20	F: 5′ TTGGACAGATATATAATGACATGGT 3′ R: 5′ AAGGAAAAATAACTAAATCACTTACTG 3′	258
21	F: 5′ AATTTCTCTAACATCTCCCTCT 3′ R: 5′ AGAATGCCAATGCAGTTAATATAC 3′	244
22	F: 5′ GTGTCTGAGACGGGTTGATT 3′ R: 5′ TCCTTCAGTCTCTTCGTACT 3′	331
23	F: 5′ TGCCCTTGTATTCCTAAGACTC 3′ R: 5′ ATTCCTTCCTTGTGTGTTGAT 3′	330
24	F: 5′ GTCTGGTTACAGGGTGATCT 3′ R: 5′ GCTGCATAGTATTCCAACAC 3′	193
25	F: 5′ GCTTCAGTAAGAGCATCTCTAAT 3′ R: 5′ TTTAGGGGTTGGAAATACTCTG 3′	300
26	F: 5′ AAACCTAATGACCTGTCATCTC 3′ R: 5′ ATAGGGAATGGCTCTGACT 3′	270
27	F: 5′ AGGAGCAATAACTGTTTCTATTT 3′ R: 5′ AGACTTATTTGTAATGATCTAAGACT 3′	241
28	F: 5′ GCATCTTTGCATCAACTTTC 3′ R: 5′ TAACTGGTGCGTCATGT 3′	277

All the primer sequences were from 5′-3′. PCR, polymerase chain reaction; HRM, high-resolution melting; F, forward primer; R, reverse primer.

**Table II:** Primers for PCR amplification of ABCB11 for direct sequencing.

Exon	Primer sequence	Amplicon length (bp)
2	F: 5′ GACTGTGGCTTATCTTTCCTG 3′ R: 5′ CGTTACATGGATTCTAGGGAG 3′	461
3	F: 5′ GAGTAAAGGTAGCAGCACTC 3′ R: 5′ GGGGACATTTGAACCTAACC 3′	500
4	F: 5′ CGCTAGTGAACCTGAGATTG 3′ R: 5′ GATAACCATGGGCTTAGTGA 3′	519
5	F: 5′ CTCTGCCACTCAATTAAGGTG 3′ R: 5′ GAAGGAAACTTGAGGCAGAG 3′	550
6	F: 5′ GGTACCATGAGGTCTGTTTAG 3′ R: 5′ CAGACTGTAGTTCTTAGGGC 3′	431
7	F: 5′ CCTGCTGAAGGTTCTGTTTA 3′ R: 5′ ACACACCAAATTGCAGTACC 3′	543
8	F: 5′ GATCTGAGAGGCTGTTAATGC 3′ R: 5′ GTTGCTAACTGTACTCAGGA 3′	414
9	F: 5′ CCCTGGATGAAGCTTACCAT 3′ R: 5′ CATACTGCTAAAGGCTTGGG 3′	504
10	F: 5′ AGTATCGCCCTTTCAACATG 3′ R: 5′ GATGCTTTTTTCCTGAAGGC 3′	494
11	F: 5′ CCAAACAGCCAAAGAGCTAG 3′ R: 5′ AGTGTTGCTGAATTAAGGGC 3′	392
12	F: 5′ CAGAGCAACAACCAGATAAAAC 3′ R: 5′ CAACACCCGAGGATACTTTC 3′	387
13	F: 5′ TACTTCTTGGTCATGGCTCT 3′ R: 5′ GTTACCATGTAGGAAGCGTG 3′	532
14	F: 5′ GCCTCTATTTTTTCTGCCCAT 3′ R: 5′ GATGAAAGGAAACACTCATGG 3′	467
15	F: 5′ GTCTGGGGAAGGGATATTTC 3′ R: 5′ TGAGGAAGATTGTAGTCAGC 3′	491
16	F: 5′ TGTGCTGGCCTTTTCTAATG 3′ R: 5′ CAGAGTTGTTGGGAGAACAG 3′	461
17	F: 5′ TAGAATCTGCAGGACAAGTC 3′ R: 5′ TCCCCAAGAAGATGAGAAGC 3′	451
18	F: 5′ CACTCTGAATCTGGGTCCAA 3′ R: 5′ GTCTGACTTGAAACACTGCT 3′	463
19	F: 5′ ATTCAAGCCACAGCAATAGT 3′ R: 5′ CTTCTTACCCTCTGTGTGATG 3′	522
20–21	F: 5′ CACAGATCCACAGCTTACAT 3′ R: 5′ ACTGGTCCCTATTCCATAGA 3′	624
22	F: 5′ ACATTGTGAAATGCCACTGA 3′ R: 5′ AGCTTCCTTCAGTCTCTTCG 3′	465
23	F: 5′ CTTTGTATTCCCAGATGATGC 3′ R: 5′ TGATGACCCACAGAATCTTG 3′	537
24	F: 5′ CTCTCCATTTCCAGACAAGT 3′ R: 5′ CTGTGTCCATGTGTTCTGTT 3′	483
25	F: 5′ CAGAACACAAAATGGAATGTCC 3′ R: 5′ TAGAATCAGGTGAAGCAGCA 3′	539
26	F: 5′ GCCTTGGGATTGTTAGTCTG 3′ R: 5′ CTGTGGAATCATGTTGGCAT 3′	486
27	F: 5′ TGCTTCCCACATCAAATGTC 3′ R: 5′ GGTTCCACAAAGTATTGCCA 3′	490
28	F: 5′ CAGGTCGTGTTAACTGAACT 3′ R: 5′ GCTTGGATTCCGATGTAGGA 3′	448

All the primer sequences were from 5′-3′. PCR, polymerase chain reaction; F, forward primer; R, reverse primer.

**Table III:** Mutations and SNPs of the ABCB11 gene in patients.

Variant	Exon	Amino acid change	RefSNP	Patients and status	Carrier rate in control (%)
c.108T>C	4	D36D	rs3815675	Heterozygous: P7, P11, P16	-
c.270T>C	5	F90F	rs4148777	Heterozygous: P6, P13	-
c.807T>C	9	Y269Y	rs2287616	Heterozygous: P7, P11, P16	-
c.1009T>C	10	Y337H	-	Heterozygous: P5	0
c.1248C>A	12	I416I	rs183390670	Heterozygous: P13	-
c.1331T>C	13	V444A	rs2287622	Heterozygous: P1, P5, P12, P16, P17, P19 Homozygous: P2, P3, P4, P6, P7, P8, P9, P10, P11, P14, P15, P18, P20	94.5
c.1415A>G	13	Y472C	-	Heterozygous: P3	0
c.2086C>T	18	R696W	-	Heterozygous: P11	0
c.2594C>T	21	A865V	rs118109635	Heterozygous: P7, P17	-
c.2782C>T	22	R928X	-	Heterozygous: P1	0
c.2792A>C	22	Q931P	-	Heterozygous: P4	0
c.3084A>G	24	A1028A	rs497692	Heterozygous: P1, P8, P12, P13, P15, P16, P17, P20 Homozygous: P2, P3, P4, P5, P6, P7, P9, P10, P14, P18, P19	90.5
c.3392A>T	25	D1131V	-	Heterozygous: P3	0
c.3593A>G	26	H1198R	-	Heterozygous: P1	0

RefSNP refers to the reference SNP in the Single Nucleotide Polymorphism Database of NCBI. SNP, single nucleotide polymorphism.

**Table IV:** Prediction of functional consequences of missense mutations and polymorphisms in ABCB11 gene found in patients.

Variant	SIFT	PolyPhen-2	SNPs&GO	EC/EN
c.1009T>C (Y337H)	0.01	0.996	Disease	EC
c.1331T>C (V444A)	0.34	0.001	Neutral	EC
c.1415A>G (Y472C)	0	1.000	Disease	EC
c.2086C>T (R696W)	0.02	0.999	Disease	EC
c.2594C>T (A865V)	0.07	0.880	Disease	EC
c.2792A>C (Q931P)	0.02	0.178	Disease	EN
c.3392A>T (D1131V)	0	1.000	Disease	EC
c.3593A>G (H1198R)	0	1.000	Disease	EC

SIFT, Sorting Intolerant From Tolerant (mutation of residues with SIFT scores <0.05 are predicted to be deleterious); PolyPhen-2, Polymorphism Phenotyping version 2 (a score <0.2 denotes benign variants, between 0.2 and 0.85 is possibly damaging and >0.85 is highly likely damaging); SNPs&GO, a web tool to predict function of SNPs with a result of neutral or disease-related variants for human; EC, evolutionarily conserved; EN, evolutionarily non-conserved; SNP, single nucleotide polymorphism.

**Table V:** Characteristics of patients with disease-causing mutations in ABCB11.

Patient	Age of onset/gender	Symptoms	GGT (U/l)	TBA (μmol/l)	TBIL/DBIL (μmol/l)	ALT/AST (U/l)	Mutation	Mutation origin
P1	1 m/M	Persistent jaundice, hepatosplenomegaly	49	101.3	162.5/130.4	432/606	Compound heterozygous p.R928X/p.H1198R	R928X, maternal; H1198R, paternal.
P3	2 d/F	Persistent jaundice, pruritus, hepatosplenomegaly	32	256.1	166.7/137.4	158/235	Compound heterozygous p.Y472C/p.D1131V	Y472C, paternal. D1131V, maternal.
P5	6 d/M	Progressive jaundice	46	NA	99.7/72.6	165/211	Heterozygous p.Y337H	NA
P11	4 d/M	Progressive jaundice	74	204.6	75.4/54.3	481/600	Heterozygous p.R696W	NA

GGT, gamma-glutamyltransferase; TBA, total bile acid; TBIL, total bilirubin; DBIL, direct bilirubin; ALT, alanine transaminase; AST, aspartate transaminase; M, male; F, female; NA, not available.

**Table VI:** Distribution of polymorphisms and allele frequencies in Chinese patients and control subjects.

A, p.V444A (c.1331T>C)						

Variable	PFIC2 (%)	Cholestasis (non-PFIC2) (%)	Control (%)	P^a^	P^b^	P^c^
Polymorphism				0.847	0.493	0.580
TT	0 (0.0)	1 (6.3)	11 (5.5)			
TC	2 (50.0)	4 (25.0)	80 (40.0)			
CC	2 (50.0)	11 (68.7)	109 (54.5)			
Total no. of patients	4	16	200			
Allele frequency				0.974	0.396	0.693
T (%)	2 (25.0)	6 (18.8)	102 (25.5)			
C (%)	6 (75.0)	26 (81.2)	298 (74.5)			

B, p.A1028A (c.3084A>G)						

Variable	PFIC2 (%)	Cholestasis (non-PFIC2) (%)	Control (%)	P^a^	P^b^	P^c^

Polymorphism				0.500	0.361	0.116
AA	1 (25.0)	0 (0.0)	19 (9.5)			
AG	1 (25.0)	7 (43.8)	93 (46.5)			
GG	2 (50.0)	9 (56.2)	88 (44.0)			
Total no. of patients	4	16	200			
Allele frequency				0.777	0.204	0.361
A (%)	3 (37.5)	7 (21.9)	131 (32.8)			
G (%)	5 (62.5)	25 (78.1)	269 (67.2)			

P^a^, PFIC2 vs. control; P^b^, cholestasis (non-PFIC2) vs. control; P^c^, PFIC2 vs. cholestasis (non-PFIC2). P<.017 was considered significant according to Bonferroni correction for multiple testing. The genotype distribution of control subjects was in Hardy-Weinberg equilibrium (V444A, P=0.46; A1028A, P=0.43). PFIC2, progressive familial intrahepatic cholestasis type 2.

## Abbreviations

PFIC2: progressive familial intrahepatic cholestasis type 2
HRM: high-resolution melting curves
SNPs: single nucleotide polymorphisms
BSEP: bile salt export pump
GGT: gamma-glutamyltransferase
